# ﻿Unveiling species diversity within early-diverging fungi from China V: Five new species of *Absidia* (Cunninghamellaceae, Mucoromycota)

**DOI:** 10.3897/mycokeys.117.149185

**Published:** 2025-05-09

**Authors:** Xin-Yu Ji, Zi-Ying Ding, Yong Nie, Heng Zhao, Shi Wang, Bo Huang, Xiao-Yong Liu

**Affiliations:** 1 College of Life Sciences, Shandong Normal University, Jinan, 250358, China Shandong Normal University Jinan China; 2 School of Civil Engineering and Architecture, Anhui University of Technology, Ma,anshan, Anhui, 243002, China Anhui University of Technology Anhui China; 3 Anhui Provincial Key Laboratory for Microbial Pest Control, Anhui Agricultural University, Hefei, Anhui, 230036, China Anhui Agricultural University Hefei China; 4 State Key Laboratory of Mycology, Institute of Microbiology, Chinese Academy of Sciences, Beijing, 100101, China Chinese Academy of Sciences Beijing China

**Keywords:** Fungal diversity, molecular phylogeny, Mucorales, soil-borne fungi, taxonomy

## Abstract

*Absidia* is widely distributed in soil, koji, and various types of feces. A multi-locus phylogeny covering the small subunit (SSU), internal transcribed spacer (ITS), and large subunit of ribosomal RNA gene (LSU rDNA), translation elongation factor 1-alpha (*TEF1α*), and actin (*Act*), combined with morphological characteristics, revealed five new species in this genus. This study provides their descriptions and illustrations and discusses their differences from morphological allies and phylogenetic relatives. *Absidiacollariata***sp. nov.** is distinguished from other species in terms of the length of collars. *A.hainanensis***sp. nov.** is named after the geographical location Hainan, distinctive with a higher maximum growing temperature. *A.pyriformis***sp. nov.** is different from other species in terms of sporangial shape. *A.tardiva***sp. nov.** is characterized by slow growth. And *A.tibetensis***sp. nov.** is named after the geographical location Tibet and differentiated by more ampulliform swellings. This study further enriches the species diversity of *Absidia* as the latest discovery of early-diverging fungi in China.

## ﻿Introduction

*Absidia* Tiegh. belongs to Mucoromycota, Mucoromycotina, Mucoromycetes, Mucorales, and Cunninghamellaceae (http://www.indexfungorum.org/, accessed on 1 November 2024). This genus was founded in 1876 and typified by *A.reflexa* Tiegh (Tieghem 1878). This genus is ubiquitous and widely distributed (Zhang et al. 2018; Crous et al. 2020; Hurdeal et al. 2021; Urquhart and Idnurm 2021; Zhao et al. 2021b; Zhao et al. 2022a); for example, the Czech Republic (688 records), Australia (862), Estonia (1621), Lithuania (422), Italy (372), South Africa (198), and Argentina (515) (https://www.gbif.org/, accessed on 2 November 2024). *Absidia* are most frequently encountered in July, August, and September, and relatively high temperatures favor *Absidia* members (https://www.gbif.org/, accessed on 2 November 2024). In China, they are mainly found in tropical regions such as Yunnan and Hainan provinces. They are occasionally isolated in places with low temperatures, such as Tibet and Jilin (Zhao et al. 2022a). The species of *Absidia* are usually isolated mainly in soil samples. They are also found in plant debris, herbivorous feces, decaying substrates, and air (Lima et al. 2020). The genus *Absidia* is very important in industry and medicine because it produces steroids, laccases, fatty acids, and other useful substances (Davoust and Persson 1992; Kristanti et al. 2016; Zhao et al. 2021b). *Absidia* is also frequently used in biotransformation of various natural products, including reduction reactions, hydroxylation, and glycosylation (Zhao et al. 2022a). Moreover, *Absidia* plays a vital role as the causative agent for human mucormycoses (Constantinides et al. 2008). And it is a contaminant in wine production (Zhao et al. 2022a).

Currently, there are 139 records of *Absidia* species, variants, and subspecies in the Index Fungorum database (http://www.indexfungorum.org/, accessed November 2, 2024). *Absidia* usually produces erect or slightly bent sporangia. There is a septum under sporangia. The sporangia are mostly nearly spherical to pyriform, deliquescent-walled, and multi-spored. Sporangiophores arise singly or in whorls. A small protuberance sometimes appears at the apex of columellae. Collars are evident if present. And zygospores have many appendages (Tieghem 1878; Guan et al. 2021; Zhao et al. 2021b; Zong et al. 2021).

Over the past few years, *Absidia* has experienced a rapid influx of proposed new species. (Lima et al. 2020; Zong et al. 2021; Cordeiro et al. 2020; Zhao et al. 2023). In this paper, five new species, *A.collariata* sp. nov., *A.hainanensis* sp. nov., *A.pyriformis* sp. nov., *A.tardiva* sp. nov., and *A.tibetensis* sp. nov., are described from the soils in Yunnan Province, Hainan Province, and Tibet, based on evidence of molecular phylogeny, morphogenetic characteristics, and maximum growth temperature. This is the fifth report of a serial work on the diversity of Chinese early-diverging fungi (Tao et al. 2024; Wang et al. 2024; Zhao et al. 2024; Ding et al. 2025).

## ﻿Materials and methods

### ﻿Isolation and morphology

In 2024, soil samples were collected from Yunnan, Tibet, and Hainan in China, following the methods of Yu Li and his colleagues (Liu et al. 2019; Zou et al. 2022). Each sample (approximately 50 g) was placed in a sterile Whirl-Pack plastic bag, labeled with a number, date, vegetation type, altitude, latitude, and longitude. All samples were preserved at 4 °C upon delivery to the laboratory. By combining soil dilution plate and moist-chamber culture methods, pure strains were isolated from soil samples (Constantinides et al. 2008; Kristanti et al. 2016; Zhao et al. 2021a). About 1 g of soil was put into a 10 mL centrifuge tube with 10 mL of sterile water and shaken on a shaker for 25 minutes to prepare a soil suspension. One milliliter of the starting suspension was transferred into 9 mL of sterile water to obtain a 10^-2^ soil suspension. The above steps were repeated to get 10^-3^ and 10^-4^ soil suspensions. Approximately 200 μL of the 10^-3^ and 10^-4^ soil suspensions were pipetted onto the Rose Bengal Chloramphenicol agar (RBC: peptone 5.00 g/L, KH_2_PO_4_ 1.00 g/L, MgSO_4_·7H_2_O 0.50 g/L, Rose Bengal 0.05 g/L, glucose 10.00 g/L, chloramphenicol 0.10 g/L, agar 15.00 g/L) and dispersed evenly with a sterilized triangular glass coating rod. The plates were cultivated at 26 °C in the dark for 2–5 days (Corry et al. 1995). Subsequently, the hyphae at the edge of the colony were transferred to a potato dextrose agar (PDA: glucose 100 g, potato 1000 g, agar 100 g, sterilized water 5000 mL, and pH 7). Upon colony forming, a macro shot was taken with a digital camera (Canon PowerShot G7X, Canon, Tokyo, Japan). For the moist chamber, 1 g of soil was evenly sprinkled onto the surface of PDA plates, sealed with Parafilm, and cultivated inverted at 26 °C away from light. After three days, the purification of target strains was performed using an inoculation ring streak. After two days, the agar with hyphae located at the edge of the colony was transferred to a new potato dextrose agar and cultivated as above. Microscopic morphological characteristics of fungi were observed with a stereoscope (SZX10, OLYMPUS, Tokyo, Japan) and a light microscope (BX53, OLYMPUS, Tokyo, Japan) and photographed with a high-definition color digital camera (DP80, OLYMPUS, Tokyo, Japan). Structural measurements were performed using Digimizer software (v5.6.0), and each trait (sporangiospores, stolons, apophyses, and so on) was measured 25 or more times. A gradient method was used to determine its maximum growth temperature (Hoffmann et al. 2007; Hoffmann and Voigt 2009; Hoffmann 2010). Cultured colonies were incubated at 25 °C for two days, and then the temperature was increased by one degree Celsius per day until no further growth was observed. This temperature was recorded as the maximum growth temperature. All strains were stored in 10% sterilized glycerin at -20 °C and preserved in the Shandong Normal University Culture Collection (XG). The living ex-holotype cultures were stored in the China Microbiological Culture Collection Center, Beijing, China (CGMCC). Dry cultures of holotypes were submitted to the Herbarium Mycologicum Academiae Sinicae, Beijing, China (HMAS). The taxonomic information was uploaded to the Fungal Names repository (https://nmdc.cn/fungalnames/).

### ﻿DNA extraction, PCR amplification, and sequencing

Fungal genomic DNA was extracted using a DNA extraction kit (Cat. No.: 70409-20; BEAVER Biomedical Engineering Co., Ltd.) (Doyle et al. 1990; Wang et al. 2023). The ITS, LSU, *TEF1α*, *Act*, and SSU regions were amplified using the primer pairs and polymerase chain reaction (PCR) programs specified in Table 1. The final volume of the reaction mixture was 25 μL, containing 12.5 μL 2 × Hieff Canace Plus PCR Master Mix with dye (Yeasen Biotechnology, Cat. No. 10154ES03), 9.5 μL ddH_2_O, 1 μL forward primer (10 μM), 1 μL reverse primer (10 μM), and 1 µL of genomic DNA template (1 ng/μL). The PCR programs are listed in Table 1. PCR amplification products were observed on a 2% agarose electrophoresis gel. Fragments were visualized at 254 nm UV light (Zhang et al. 2022). The amplified product was purified using a gel extraction kit (Cat# AE0101-C, Shandong Sparkjade Biotechnology Co., Ltd.). Primer synthesis and DNA sequencing were carried out by Tsingke Biotechnology (Beijing, China). MEGA v. 7.0 (Mega Limited, Auckland, New Zealand) was used to obtain consensus sequences. Finally, all sequences generated in this study were deposited in GenBank.

**Table 1.** PCR information used in this study.

Loci	PCR primers	Primer sequence (5’ – 3’)	PCR cycles	References
ITS	ITS5	GGA AGT AAA AGT CGT AAC AAG G	95 °C 5 min; (95 °C: 30 s, 55 °C: 30 s, 72 °C: 1 min) × 35 cycles; 72 °C 10 min	White et al. (1990)
	ITS4	TCC TCC GCT TAT TGA TAT GC		
LSU	LR0R	GTA CCC GCT GAA CTT AAG C	95 °C 5 min; (94 °C: 30 s, 52 °C: 45 s, 72 °C: 1.5 min) × 30 cycles; 72 °C 10 min	Hurdeal et al. (2023)
	LR5	TCC TGA GGG AAA CTT CG		
*TEF1α*	EF1-983F	GCYCCYGGHCAYCGTGAYTTYAT	95 °C 5 min; (95 °C: 30 s, 55 °C: 60 s, 72 °C: 60 s) × 30 cycles; 72 °C 10 min	Jaklitsch et al. (2005)
	TEF1LLErev	AACTTGCAGGCAATGTGG		
*Act*	ACT-1	TGG GAC GAT ATG GAI AAI ATC TGG CA	95 °C 3 min; (95 °C: 60 s, 55 °C: 60 s, 72 °C: 1 min) × 30 cycles; 72 °C 10 min	Voigt and Wöstemeyer (2000)
	ACT-4R	TC ITC GTA TIC TIG CTI IGA IAT CCA CA T		
SSU	NS1	GTA GTC ATA TGC TTG TCT CC	95 °C 5 min; (94 °C: 60 s, 54 °C: 50 s, 72 °C: 1 min) × 37 cycles; 72 °C 10 min	(White et al. 1990)
	NS4	CTT CCG TCA ATT CCT TTA AG		

### ﻿Phylogenetic analyses

Reference sequences were downloaded according to the latest articles (Zhao et al. 2021b; Zhao et al. 2022a; Tao et al. 2024; Zhao et al. 2024). Phylogenetic analyses were performed for each marker individually, followed by a combined analysis (ITS-LSU-SSU-*Act*-*TEF1α*). The sequences newly obtained in this study were compared with reference sequences in GenBank using MEGA v.7.0 software (Larsson 2014; Kumar et al. 2016). The phylogeny was inferred using maximum likelihood (ML) and Bayesian inference (BI) algorithms (Nie et al. 2020a; Nie et al. 2020b), which were integrated with the CIPRES Science Portal (https://www.phylo.org/, accessed November 5, 2024). ML analysis was performed using RaxML 8.2.4 (https://www.phylo.org/) in CIPRES Science Gateway V. 3.3 with 1,000 bootstrap replicates (Miller et al. 2010; Nguyen et al. 2015). BI analysis was performed using the GTR + I + G model with a sampling frequency of every 1,000 generations, and eight cold Markov chains were run simultaneously for two million generations (Ronquist et al. 2012; Stamatakis 2014). Utilizing the iTOL website (https://itol.embl.de, accessed November 5, 2024), all trees that resulted were plotted and optimized. Finally, Adobe Illustrator CC 2019 was used to beautify the phylogenetic tree (Jiang et al. 2024).

## ﻿Results

### ﻿Molecular phylogeny

**Figure 1.**
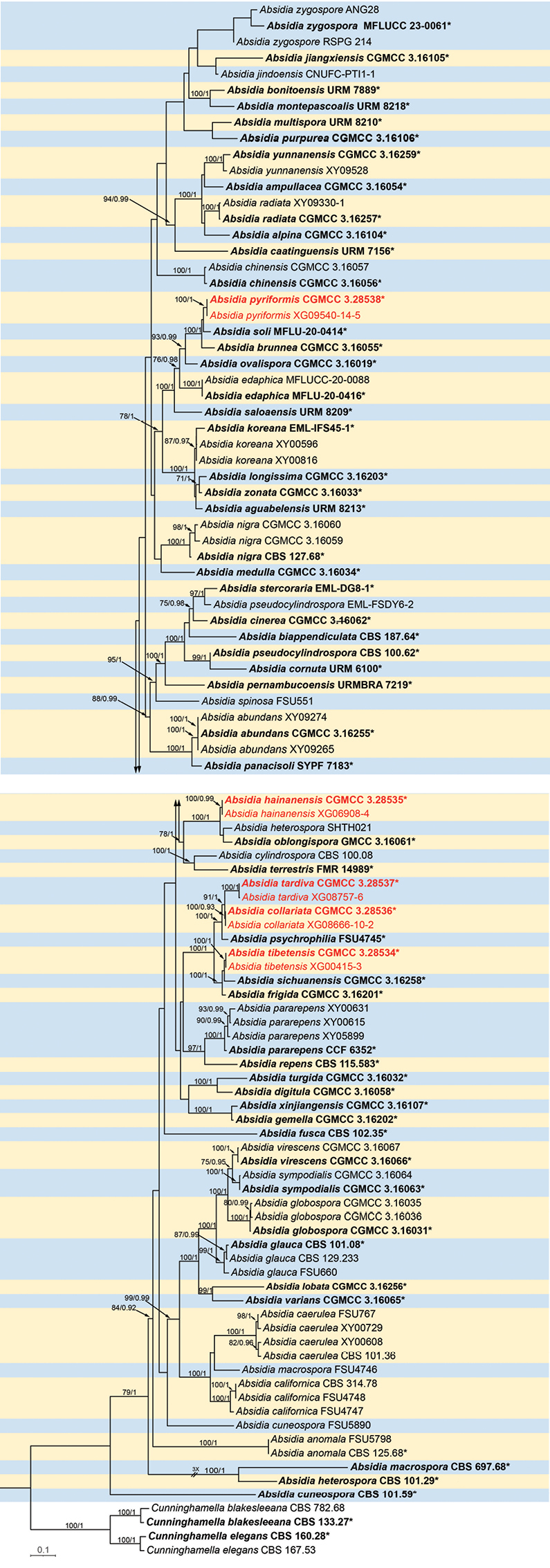
Phylogram of the genus *Absidia* based on a concatenated ITS, LSU, *TEF1α*, *Act*, and SSU sequence alignment, with *Cunninghamellaelegans* and *C.blakesleeana* serving as outgroups. The robustness of branches is marked at the node with the Maximum Likelihood Bootstrap Value (left, MLBV ≥ 70%) and Bayesian Inference Posterior Probability (right, BIPP ≥ 0.90), which are separated by a slash “/”. Ten newly isolated strains are indicated in red bold. Branches shortened to fit the page are indicated by a double slash “//”. Bold strains marked with a star marker “*” are ex-types or ex-holotypes. The scale at the bottom left indicates 0.1 substitutions per site.

Phylogenetic analyses were performed on a dataset containing 103 isolates, including 93 strains retrieved from GenBank and 10 acquired herein. Of these, 99 isolates were classified as the ingroup *Absidia*, while four strains, *Cunninghamellaelegans* (CBS 160.28), *C.elegans* (CBS 167.53), *C.blakesleeana* (CBS 133.27), and *C.blakesleeana* (CBS 782.68), were employed as outgroups. In total, it consisted of 5,087 concatenated characters: 1–1,102 (ITS), 1,103–2,072 (LSU), 2,073–3,157 (*TEF1α*), 3,158–4,118 (*Act*), and 4,119–5,087 (SSU). Among these, 2,766 characters remained constant, 724 were variable and parsimony-uninformative, and 1,597 were parsimony-informative. The maximum likelihood (ML) tree (Fig. 1) and Bayesian tree showed comparable topological structures.

### ﻿Taxonomy

#### ﻿ Absidia collariata

Taxon classificationFungiMucoralesCunninghamellaceae

X.Y. Ji, H. Zhao & X.Y. Liu sp. nov.

9DD6FFC5-9E10-59D9-AF8E-C3F8BCBFE9CD

Fungal Names: FN 572256

Fig. 2

##### Type.

China • Yunnan Province, Yuxi City, Xinping Yi Dai Autonomous County, Ancient Tea Horse Road (23°57'28"N, 101°30'38"E, 2196.56 m), from soil, 5 Jul. 2024, X.Y. Ji and X.Y. Liu, holotype HMAS 353360, ex-holotype living culture CGMCC 3.28536 (=XG08666-10-1).

**Figure 2.**
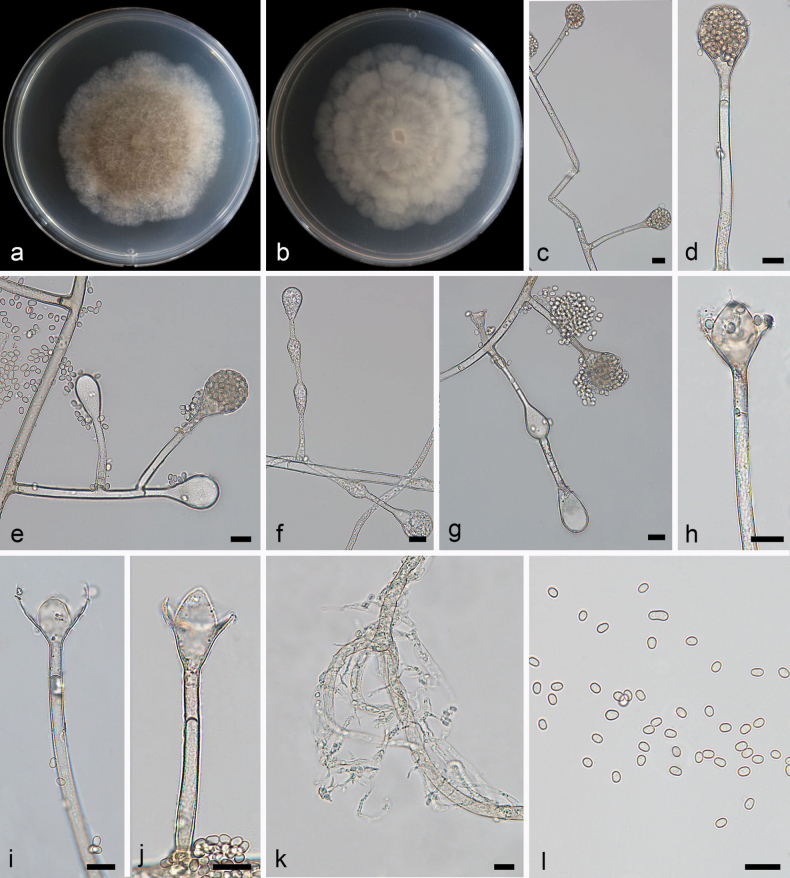
*Absidiacollariata* ex-holotype CGMCC 3.28536 **a, b** colonies on PDA (**a** obverse **b** reverse) **c–e** sporangia **f, g** swelling on sporangiophores **h–j** columellae **k** rhizoids **l** sporangiospores. Scale bars: 10 μm (**c–l**).

##### Etymology.

The *collariata* (Lat.) refers to its long collars.

##### Description.

Colonies on PDA at 26 °C for 5 days, reaching 68 mm in diameter, moderately fast growing with a rate of 13.6 mm/d, higher in the center than at margin, at first white, becoming grayish brown when mature, regular shape at reverse. Hyphae light-colored at first, becoming brown when mature, 4.7–9.4 µm (x– = 6.4 µm, n = 20) wide. Stolons branched, hyaline to light brown, smooth, septate, 5.1–6.3 µm (x– = 5.6 µm, n = 15) in diameter. Rhizoids well developed, root-like, branched. Sporangiophores growing on stolons, erect or slightly bent, mostly unbranched or simply branched, smooth, single or 2–4 in whorls, monopodial or sympodial, 21.7–213.5 × 2.3–5.4 µm (x– = 112.8 × 4.1 µm, n = 15). Sporangia globose to pyriform, smooth, hyaline, deliquescent-walled, 16.2–37.0 × 14.0–31.1 µm (x– = 24.2 × 23.2 µm, n = 15), and with a septum 8.8–17.9 µm (x– = 13.9 µm, n = 15) below apophyses; the septum is not obvious when young. Apophyses distinct, funnel-shaped, 6.0–9.5 µm (x– = 8.5 µm, n = 15) high, 5.1–8.8 µm (x– = 6.2 µm, n = 15) wide at the base, and 8.4–13.9 µm (x– = 10.7 µm, n = 15) wide at the top, darker brown when old. Collars present, obvious. Columellae nearly conical, sometimes subspherical to hemispherical, 8.1–13.8 × 7.7–14.9 µm (x– = 10.6 × 10.9 µm, n = 15). Projections absent or present, hyaline, single. Sporangiospores hyaline, smooth, mostly oval, 2.1–3.8 × 1.8–2.7 µm (x– = 3.1 × 2.3 µm, n = 20). Chlamydospores absent. Zygospores not found.

##### Maximum growth temperature.

29 °C.

##### Additional specimen examined.

China • Yunnan Province, Yuxi City (23°57'28"N, 101°30'38"E, 2196.56 m), from soil, 5 Jul. 2024, X.Y. Ji and X.Y. Liu, living culture XG08666-10-2.

##### Notes.

In the molecular phylogeny, *A.collariata* was closely related to *A.psychrophilia* (Zhao et al. 2022b). Morphologically, the width of stolons in *A.collariata* was smaller than that in *A.psychrophilia* (5.1–6.3 µm vs. 5.5–11 µm). Furthermore, the *A.collariata* has smaller sporangiophores (2.1–3.8 × 1.8–2.7 µm vs. 3.8–5 × 2.2–3.5 µm). The length and width of sporangiophore were also smaller in *A.collariata* than those in *A.psychrophilia* (21.7–213.5 × 2.3–5.4 µm vs. 193–288 × 4.5–9 µm). And the *A.psychrophilia* has larger sporangia (20–50 μm vs. 16.2–37.0 × 14.0–31.1 μm). Zygospores were not observed in *A.collariata*.

#### ﻿ Absidia hainanensis

Taxon classificationFungiMucoralesCunninghamellaceae

X.Y. Ji, H. Zhao & X.Y. Liu sp. nov.

794BA142-C23B-5F4D-967D-1F915419A3BB

Fungal Names: FN 572258

Fig. 3

##### Type.

China • Hainan Province, Danzhou City, Tropical Botanical Garden (19°30'42"N, 109°30'3"E, 168.7 m), from soil, 26 Jun. 2024, X.Y. Ji and X.Y. Liu, holotype HMAS 353362, ex-holotype living culture CGMCC 3.28535 (=XG06908-1).

**Figure 3.**
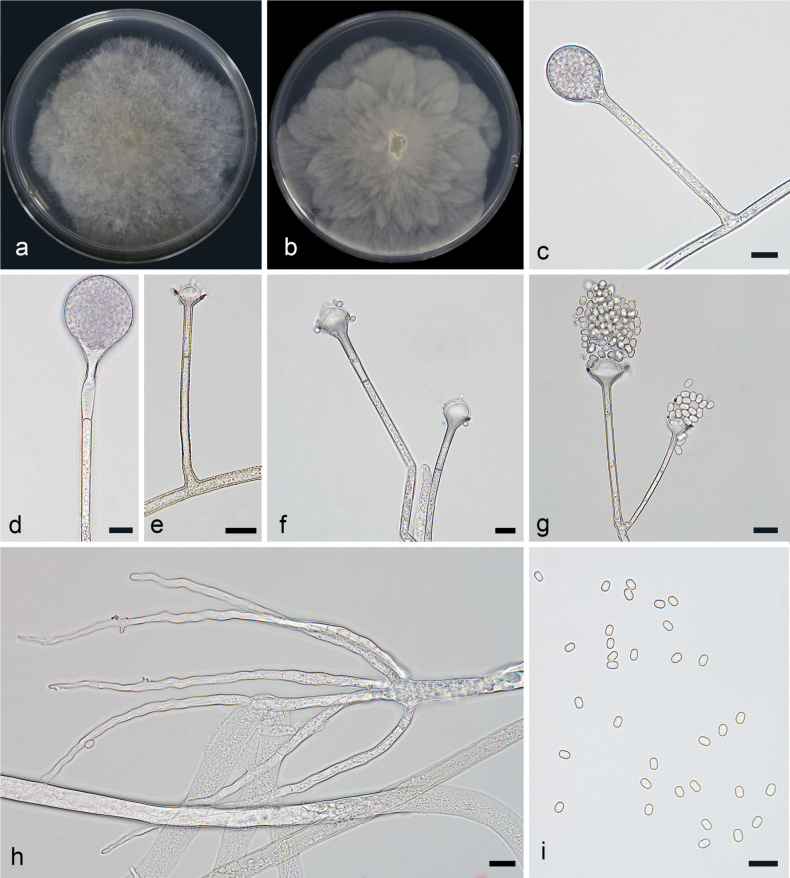
*Absidiahainanensis* ex-holotype CGMCC 3.28535 **a, b** colonies on PDA (**a** obverse **b** reverse) **c, d** sporangia **e–g** columellae **h** rhizoids **i** sporangiospores; Scale bars: 10 µm (**c–i**).

##### Etymology.

The *hainanensis* (Lat.) refers to Hainan Province of China, where the type was collected.

##### Description.

Colonies on PDA at 26 °C for 5 days, reaching 75 mm in diameter, fast growing with a rate of 15 mm/d, at first white, becoming grayish-brown when old. Hyphae hyaline at first, becoming light brown when mature, 2.5–10.2 µm (x– = 5.2 µm, n = 20) in diameter. Rhizoids root-like, simply branched. Stolons hyaline, smooth, branched, 2.9–10.1 µm (x– = 6.1 µm, n = 15) in diameter. Sporangiophores erect or slightly bent, mostly unbranched or simply branched, smooth, monopodial or sympodial, single or 2–4 in whorls, 18.8–159.2 × 1.9–3.7 µm (x– = 75.4 × 2.8 µm, n = 15). Sporangia spherical to subspherical, smooth, hyaline, deliquescent-walled, 15.1–35.2 × 14.3–29.4 µm (x– = 24.2 × 22.1 µm, n = 15), and with a septum 11.5–24.2 µm (x– = 17.0 µm, n = 15) below apophyses. Apophyses obvious, funnel-shaped, 3.3–5.1 µm (x– = 4.6 µm, n = 15) high, 2.5–8.5 µm (x– = 4.3 µm, n = 15) wide at the base, and 7.5–17.4 µm (x– = 11.0 µm, n = 15) wide at the top, light brown, hyaline. Collars present. Columellae mostly oval, 3.9–13.9 × 8.6–19.5 µm (x– = 6.3 × 10.7 µm, n = 15). Projections absent or present, hyaline, single. Sporangiospores ovoid to cylindrical, smooth, hyaline, 3.1–3.7 × 1.9–2.5 µm (x– = 3.6 × 2.2 µm, n = 20). Chlamydospores absent. Zygospores not found.

##### Maximum growth temperature.

34 °C.

##### Additional specimen examined.

China • Hainan Province, Danzhou City (19°30'42"N, 109°30'3"E, 168.7 m), from soil, 26 June 2024, X.Y. Ji and X.Y. Liu, living culture XG06908-4.

##### Notes.

In the molecular phylogeny, *A.hainanensis* was closely related to *A.oblongispora* (Zong et al. 2021). Morphologically, the sporangiophores of *A.hainanensis* were at most four in whorls, while those of *A.oblongispora* were at most five in whorls. Additionally, the maximum length of the sporangiophores in *A.oblongispora* was significantly greater than that in *A.hainanensis* (300 µm vs. 159.2 µm). The sporangiospore size was smaller in *A.hainanensis* (3.1–3.7 × 1.9–2.5 μm vs. 3.5–4.5 × 2.5–8.5 μm). The *A.hainanensis* has wider columellae (8.6–19.5 µm vs. 8.5–16.5 µm). Physiologically, the maximum growth temperature of *A.hainanensis* was higher (34 °C vs. 32 °C).

#### ﻿ Absidia pyriformis

Taxon classificationFungiMucoralesCunninghamellaceae

X.Y. Ji, H. Zhao & X.Y. Liu sp. nov.

28DB24F4-CF53-5C0A-956D-2E42209C3DDA

Fungal Names: FN 572255

Fig. 4

##### Type.

China, Yunnan Province, Pu’er City, Mojiang Hani Autonomous County, Lianzhu Town (23°25'34"N, 101°40'58"E, 1338.32 m), from soil, 4 July 2024, X.Y. Ji and X.Y. Liu, holotype HMAS 353359, ex-holotype living culture CGMCC 3.28538 (=XG09540-14-1).

**Figure 4.**
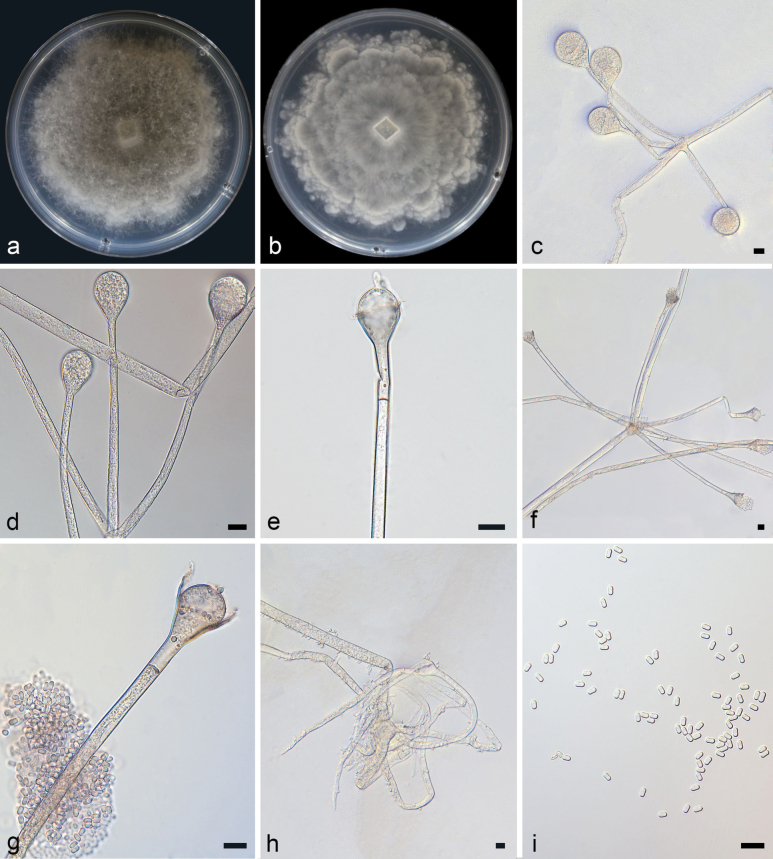
Absidiapyriformis ex-holotype CGMCC 3.28538 **a, b** colonies on PDA (**a** obverse **b** reverse) **c, d** sporangia **e–g** columellae **h** rhizoids **i** sporangiospores; Scale bars: 10 µm (**c–i**).

##### Etymology.

The epithet *pyriformis* (Lat.) refers to the shape of the sporangia.

##### Description.

Colonies on PDA at 26 °C for 5 days, attaining 76 mm in diameter, moderately fast growing with a rate of 15.2 mm/d, white at first, gradually light gray, irregularly at reverse. Hyphae branched, hyaline at first, sometimes brownish when mature, aseptate when juvenile, septate with age, 3.6–15.2 µm (x– = 6.3 µm, n = 20) wide. Stolons branched, hyaline, smooth, septate, 4.5–11.9 µm (x– = 6.9 µm, n = 15) in diameter. Rhizoids well developed, root-like, branched, tapering at the end. Sporangiophores arising from stolons, erect or slightly bent, 2–5 in whorls, monopodial, mostly unbranched or simply branched, smooth, 21.7–279.8 × 1.4–7.4 µm (x– = 97.9 × 4.4 µm, n = 15), with one septum 11.5–26.8 µm (x– = 18.5 µm, n = 15) below apophyses. Sporangia are mostly pyriform, deliquescent-walled, smooth, multi-spored, colorless when young, brownish when old, 11.7–38.8 × 11.0–29.7 µm (x– = 27.9 × 22.1 µm, n = 15). Apophyses distinct, subhyaline, usually brownish when old, 5.0–8.4 µm (x– = 7.0 µm, n = 15) high, 2.4–6.1 µm (x– = 4.4 µm, n = 15) wide at the base, and 8.9–19.2 µm (x– = 12.5 µm, n = 15) wide at the top. Collars distinct. Columellae hemispherical, subglobose to globose, smooth, subhyaline or brownish, 8.8–21.4 × 16.7–20.7 µm (x– = 10.9 × 16.7 µm, n = 15). Projections at the apex, when smaller, with an oval projection. Sporangiospores hyaline, smooth, almost cylindrical, 3.2–4.5 × 1.7–2.8 µm (x– = 3.9 × 2.2 µm, n = 20). Chlamydospores absent. Zygospores absent.

##### Maximum growth temperature.

33 °C.

##### Additional specimen examined.

China • Yunnan Province, Pu’er City, from soil (23°25'34"N, 101°40'58"E, 1338.32 m), 4 July 2024, X.Y. Ji and X.Y. Liu, living culture XG09540-14-5.

##### Notes.

Phylogenetically, *A.pyriformis* was closely related to *A.soli* (Hurdeal et al. 2021). Compared with *A.soli*, the *A.pyriformis* presented a smaller sporangia size (11.7–38.8 × 11.0–29.7 µm vs. 16–51 × 15–45.5 µm), and the septum showed at a shorter distance from apophyses (11.5–26.8 µm vs. 21.5–37.5 µm); conversely, sporangiophores exhibited a larger size (3.2–4.5 µm vs. 1.7–2.8 µm), and columellae had a longer length (8.8–21.4 µm vs. 7.5–12.5 µm).

#### ﻿ Absidia tardiva

Taxon classificationFungiMucoralesCunninghamellaceae

X.Y. Ji, H. Zhao & X.Y. Liu sp. nov.

0C92F093-24E0-51BB-B154-B0292C9C8216

Fungal Names: FN 572254

Fig. 5

##### Type.

China, Yunnan Province, Yuxi County, Jinshan National Forest (23°38'15"N, 101°16'30"E, 2397.53 m), from soil, 14 May 2024, X.Y. Ji and X.Y. Liu, holotype HMAS 353358, ex-holotype living culture CGMCC 3.28537 (=XG08757-4).

**Figure 5.**
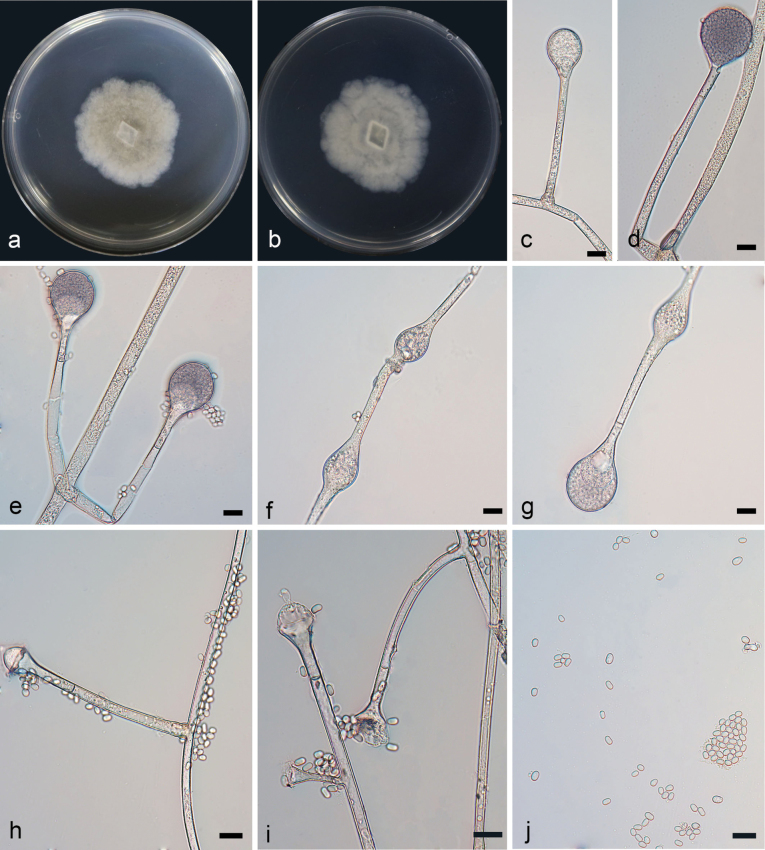
*Absidiatardiva* ex-holotype CGMCC 3.28537 **a, b** colonies on PDA (**a** obverse **b** reverse) **c, e** sporangia **f, g** a swelling on sporangiophores and hyphae **h, i** columellae **j** sporangiospores. Scale bars: 10 µm (**c–j**).

##### Etymology.

The epithet *tardiva* (Lat.) refers to this species growing more slowly than other strains.

##### Description.

Colonies on PDA at 26 °C for 4 days, reaching 41 mm in diameter, slow-growing with a rate of 10.25 mm/d; it begins white and gradually turns pale yellow to grayish-brown, irregular at reverse. Hyphae branched, hyaline at first, brownish when mature, 2.6–10.7 µm (x– = 5.6 µm, n = 20) in diameter, sometimes swollen. Stolons hyaline to brownish, smooth, branched, 3.7–8.4 µm (x– = 5.7 µm, n = 15) in diameter. Rhizoids not observed. Sporangiophores erect or slightly bent, single or 2–4 in whorls, unbranched or simply branched, monopodial or sympodial, with a septum 10.6–23.1 µm (x– = 13.8 µm, n = 15) below apophyses, 7.9–141.9 × 1.9–7.4 µm (x– = 70.7 × 4.2 µm, n = 15), sometimes with a swelling beneath sporangia. Sporangia subspherical to spherical, smooth, multi-spored, 12.9–48.3 × 9.3–34 µm (x– = 30.3 × 22.2 µm, n = 15), deliquescent-walled. Apophyses distinct, subhyaline, small, slightly pigmented, 3.2–10.4 µm (x– = 5.2 µm, n = 15) high, 2.9–7.2 µm (x– = 4.4 µm, n = 15) wide at the base, and 8.0–20.5 µm (x– = 13.2 µm, n = 15) wide at the top. Collars absent. Columellae hemispherical, subhyaline to hyaline, smooth, 2.9–13.8 × 4.9–16.3 µm (x– = 8.7 × 9.8 µm, n = 15). Projections present, shaped like a grain of rice. Sporangiospores variously shaped, mostly ovoid; a few are cylindrical or subglobose, smooth, hyaline, 3.4–4.6 × 2.1–2.8 µm (x– = 3.9 × 2.3 µm, n = 20). Chlamydospores absent. Zygospores not observed.

##### Maximum growth temperature.

27 °C.

##### Additional specimen examined.

China • Yunnan Province, Yuxi County, from soil (23°38'15"N, 101°16'30"E, 2397.53 m), 14 May 2024, X.-Y. Ji and X.-Y. Liu, living culture XG08757-6.

##### Notes.

Phylogenetic analysis of five genes showed that *A.tardiva* was closely related to *A.psychrophilia* (Zhao et al. 2022b). Morphologically, the sporangia shape of *A.psychrophilia* was pyriform, while the sporangia of *A.tardiva* were hemispherical to spherical in shape. And the distance between the septum and apophysis was shorter in *A.psychrophilia* than in *A.tardiva* (10–17 µm vs. 10.6–23.1 µm). The columellae of *A.psychrophilia* were larger than those of *A.tardiva* (6.5–30 μm in diameter vs. 2.9–13.8 × 4.9–16.3 μm). The overall size of spores in *A.psychrophilia* was slightly larger than that in *A.tardiva* (long: 3.8–5 × 2.2–3.5 µm vs. wide: 3.4–4.6 × 2.1–2.8 µm); the shape of sporangiospores in *A.psychrophilia* was cylindrical, whereas the shape of spores in *A.tardiva* was oval.

#### ﻿ Absidia tibetensis

Taxon classificationFungiMucoralesCunninghamellaceae

X.Y. Ji, H. Zhao & X.Y. Liu sp. nov.

5320FDCE-5F06-5C53-BD2F-13E046DD1D8F

Fungal Names: FN 572257

Fig. 6

##### Type.

China • Tibet, Xigaze City, Yadong Country (27°21'53"N, 88°58'26"E, 2827 m), from soil, 1 Oct 2024, X.Y. Ji and X.Y. Liu, holotype HMAS 353361, ex-holotype living culture CGMCC 3.28534 (=XG00415-1).

**Figure 6.**
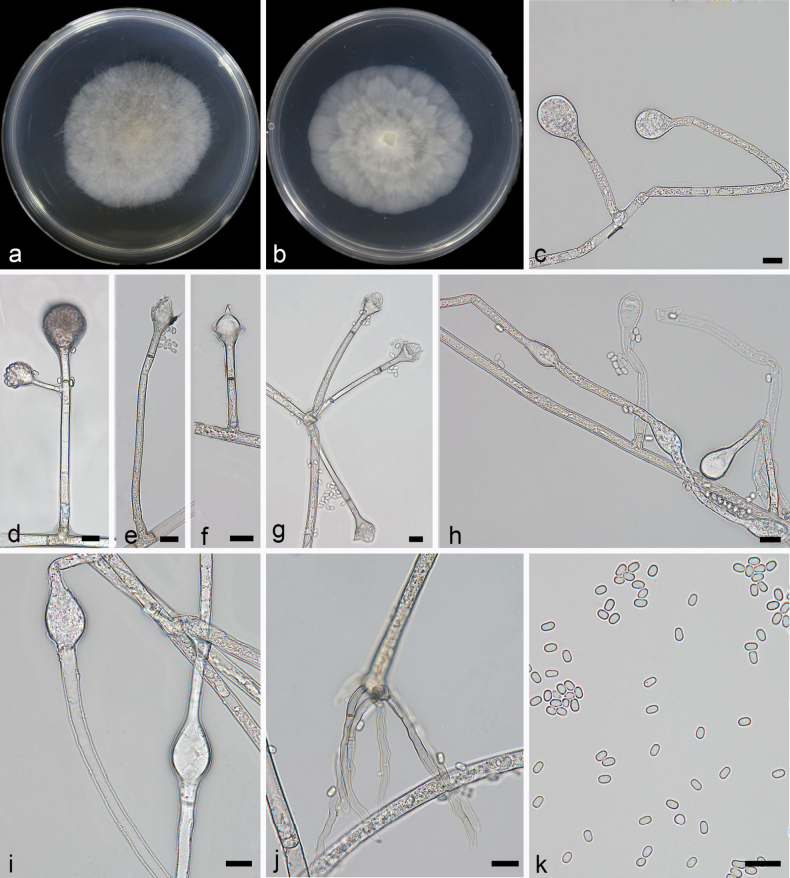
*Absidiatibetensis* ex-holotype CGMCC 3.28534 **a, b** colonies on PDA (**a** obverse **b** reverse) **c, d** sporangia **e–g** columellae **h, i** a swelling on sporangiophores and hyphae **j** rhizoids **k** sporangiospores. Scale bars: 10 μm (**c–k**).

##### Etymology.

The *tibetensis* (Lat.) refers to the Tibet Autonomous Region of China, where the type was collected.

##### Description.

Colonies on PDA at 26 °C for 5 days, reaching 53 mm in diameter, slow-growing with a rate of 10.6 mm/d, white at first and gradually turning to light brown; the reverse side of the colony resembles a petal-shaped, regularly at reverse. Rhizoids root-like, always branched, with a septum at the top. Hyphae hyaline to slightly gray, 5.0–10.0 µm (x– = 7.1 µm, n = 20) in diameter, sometimes ampulliform-shaped swollen. Stolons hyaline, slightly brownish, branched, smooth, 3.2–11.0 µm (x– = 6.0 µm, n = 15) in diameter. Sporangiophores erect or slightly bent, unbranched or simple branched, smooth, single or 2–5 in whorls, monopodial or sympodial, 14.7–144.0 × 2.5–5.7 µm (x– = 78.2 × 4.0 µm, n = 15), sometimes with a swelling beneath sporangia. Sporangia globose to pyriform, smooth, multi-spored, deliquescent-walled, 11.0–30.2 × 11.1–26.6 µm (x– = 21.5 × 17.1 µm, n = 15), and with a septum 8.4–20.0 µm (x– = 15.6 µm, n = 15) below apophyses. Apophyses obvious, funnel-shaped, gradually widening from the base to the top, 2.5–9.6 µm (x– = 6.7 µm, n = 15) high, 3.2–8.3 µm (x– = 4.2 µm, n = 15) wide at the base, and 7.4–19.0 µm (x– = 11.2 µm, n = 15) wide at the top, hyaline, light brown. Collars absent or present. Columellae conical, nearly globose, occasionally oval, 8.5–19.9 × 10.1–16.5 µm (x– = 11.3 × 11.8 µm, n = 15). Projections present or absent, hyaline when present, needle-pointed. Sporangiospores smooth, hyaline, mostly oval, 2.6–3.9 × 1.6–2.4 µm (x– = 3.6 × 2.1 µm, n = 20). Chlamydospores absent. Zygospores not found.

##### Maximum growth temperature.

30 °C.

##### Additional specimen examined.

China • Tibet, Xigaze City, Yadong County (27°21'53"N, 88°58'26"E, 2827 m), from soil, 1 October 2024, X.Y. Ji and X.Y. Liu, living culture XG00415-3.

##### Notes.

In the molecular phylogeny, *A.tibetensis* was closely related to *A.sichuanensis* (Zhao et al. 2022b). Morphologically, the maximum width of the hyphae in *A.yunnanensis* was greater than that in *A.tibetensis* (15.5 µm vs. 10.0 µm). Apophyses had a wider base width and top width in *A.tibetensis* (3.2–8.3 × 7.4–19.0 μm vs. 3.0–5.0 × 5.5–12.0 μm). The sporangiophore size was smaller in *A.tibetensis* (2.6–3.9 × 1.6–2.4 μm vs. 3.0–4.5 × 2.0–2.5 μm). The swelling on sporangiophores and hyphae was observed in *A.tibetensis*. The collars were not observed in *A.sichuanensis*. Physiologically, the maximum growth temperature of *A.tibetensis* was higher (30 °C vs. 28 °C).

## ﻿Discussion

*Absidia* is widely distributed. Some soil samples in Yunnan Province, Tibet Autonomous Region, and Hainan Province were investigated in this study. The cities Pu’er and Yuxi in Yunnan Province have a subtropical monsoon climate with complex terrain, mild and humid climate, and abundant precipitation. The climatic environment is conducive to the growth of various microorganisms. Yadong County of the Tibet Autonomous Region has a plateau and mountainous climate, with significant seasonal changes and extreme weather phenomena. Danzhou City, in Hainan Province, has a tropical humid monsoon climate with abundant sunshine and abundant rainfall. Five new species of the genus *Absidia* were discovered in these places (Wu et al. 2020; Wang et al. 2021; Zhao et al. 2022b; Jiang et al. 2024).

Based on morphology, growth temperature dynamics, and molecular phylogenetic analyses, five novel species were identified in the genus *Absidia*, namely *A.collariata* sp. nov., *A.hainanensis* sp. nov., *A.pyriformis* sp. nov., *A.tardiva* sp. nov., and *A.tibetensis* sp. nov. In this study, phylogenetic analysis was performed for these five novel species based on five loci, namely ITS, LSU, *TEF1α*, *Act*, and SSU. By analyzing these data, strong support was obtained for the clades of these species (*A.tardiva* 100% MLBV and 1.00 BIPP; *A.pyriformis* 100% MLBV and 1.00 BIPP; *A.collariata* 100% MLBV and 1.00 BIPP; *A.tibetensis* 100% MLBV and 1.00 BIPP; *A.hainanensis* 100% MLBV and 1.00 BIPP; Fig. 1). At the same time, in terms of morphological structure and physiology, we also found some differences between these five newly discovered species and their closely related species (Benny 2008; Zheng et al. 2009; Hurdeal et al. 2021; Urquhart and Idnurm 2021). They have great differences in sporangiospore size, stolon width, sporangia size, base and top width of the apophyses, and so on (Ellis and Hesseltine 1965; Davoust and Persson 1992; Lima et al. 2020). Besides, the maximum growth temperature between them is also different. These differences laid the foundation for the identification of the five novel species.

**Table 2.** GenBank accession numbers of sequences used in this study.

Species	Strains	GenBank accession numbers				
		ITS	LSU	TEF-1α	Act	SSU
*Absidiaabundans*	XY09265	ON074697	ON074681	NA	NA	NA
*A.abundans*	CGMCC 3.16255*	NR_182590	ON074683	NA	NA	NA
*A.abundans*	XY09274	ON074696	ON074682	NA	NA	NA
*A.aguabelensis*	URM 8213*	NR_189383	NG_241934	NA	NA	NA
*A.alpina*	CGMCC 3.16104	OL678133	NA	NA	NA	NA
*A.ampullacea*	CGMCC 3.16054	MZ354138	MZ350132	NA	NA	NA
*A.anomala*	CBS 125.68*	MH859085	MH870799	NA	NA	NA
*A.anomala*	FSU5798	EF030523	NA	NA	EF030535	NA
*A.biappendiculata*	CBS 187.64	MZ354153	MZ350147	MZ357420	MZ357438	NA
*A.bonitoensis*	URM 7889*	MN977786	MN977805	NA	NA	NA
*A.brunnea*	CGMCC 3.16055*	MZ354139	MZ350133	MZ357403	MZ357421	NA
*A.caatinguensis*	URM 7156*	NR_154704	NG_058582	NA	NA	NA
*A.caerulea*	XY00608	OL620081	NA	NA	NA	NA
*A.caerulea*	XY00729	OL620082	NA	NA	NA	NA
*A.caerulea*	CBS101.36	MH855718	MH867230	NA	NA	NA
*A.caerulea*	FSU767	AY944870	NA	NA	NA	NA
*A.californica*	CBS 314.78	JN205816	MH872902	NA	NA	NA
*A.californica*	FSU4748	AY944873	EU736301	EU736247	EU736224	EU736274
*A.californica*	FSU4747	AY944872	EU736300	EU736246	AY944758	EU736273
*A.chinensis*	CGMCC 3.16057	MZ354141	MZ350135	NA	MZ357422	NA
*A.chinensis*	CGMCC 3.16056*	MZ354140	MZ350134	NA	NA	NA
*A.cinerea*	CGMCC 3.16062	MZ354146	MZ350140	MZ357407	MZ357427	NA
***A.collariata***	CGMCC 3.28536*	**PQ610533**	**PQ605104**	**PQ613269**	**PQ613279**	**PQ605114**
***A.collariata***	**XG08666-10-2**	**PQ610534**	**PQ605105**	**PQ613270**	**PQ613280**	**PQ605115**
*A.cornuta*	URM 6100*	NR_172976	MN625255	NA	NA	NA
*A.cuneospora*	CBS 101.59*	MH857828	MH869361	NA	NA	NA
*A.cylindrospora*	CBS 100.08	JN205822	JN206588	NA	NA	NA
*A.digitula*	CGMCC 3.16058*	MZ354142	MZ350136	MZ357404	MZ357423	NA
*A.edaphica*	MFLUCC 20-0088	NR_172305	NG_075367	NA	MT410739	NG_074951
*A.frigida*	CGMCC 3.16201*	NR_182565	OM030223	NA	NA	NA
*A.fusca*	CBS 102.35*	NR_103625	NG_058552	NA	NA	NA
*A.gemella*	CGMCC 3.16202*	OM108488	OM030224	NA	NA	NA
*A.glauca*	CBS 129233	MH865253	MH876693	NA	NA	NA
*A.glauca*	CBS 101.08*	MH854573	MH866105	NA	NA	NA
*A.glauca*	FSU660	AY944879	EU736302	EU736248	EU736225	EU736275
*A.globospora*	CGMCC 3.16031*	NR_189829	MW671544	MZ357412	MZ357431	NA
*A.globospora*	CGMCC 3.16035	MW671538	MW671545	MZ357413	MZ357432	NA
*A.globospora*	CGMCC 3.16036	MW671539	MW671546	MZ357414	MZ357433	NA
***A.hainanensis***	CGMCC 3.28535*	**PQ610537**	**PQ605108**	**PQ613273**	**PQ613283**	**PQ605118**
***A.hainanensis***	**XG06908-4**	**PQ610538**	**PQ605109**	**PQ613274**	**PQ613284**	**PQ605119**
*A.heterospora*	SHTH021	JN942683	JN982936	NA	NA	JQ004928
*A.heterospora*	CBS101.29*	JN206595.1	MH866483.1	NA	NA	NA
*A.jiangxiensis*	CGMCC 3.16105*	OL678134	PP780377	PP790569	PP790577	PP779719
*A.jindoensis*	CNUFC-PTI1-1	MF926622	MF926616	MF926513	MF926510	MF926626
*A.koreana*	EML-IFS45-1*	KR030062	KR030056	KR030060	KR030058	KT321298
*A.koreana*	XY00816	OL620083	ON123771	NA	NA	NA
*A.koreana*	XY00596	OL620084	NA	NA	NA	NA
*A.lobata*	CGMCC 3.16256	ON074690	ON074679	NA	NA	NA
*A.longissima*	CGMCC 3.16203*	NR_182566	OM030225	NA	NA	NA
*A.macrospora*	FSU4746	AY944882	EU736303	EU736249	AY944760	EU736276
*A.macrospora*	CBS 697.68*	HM849704.1	NA	NA	NA	NA
*A.medulla*	CGMCC 3.16034	NR_189832	MW671549	MZ357417	MZ357436	NA
*A.montepascoalis*	URM 8218	NR_172995	NA	NA	NA	NA
*A.multispora*	URM 8210*	MN953780	MN953782	NA	NA	NA
*A.nigra*	CBS 127.68*	NR_173068	MZ350146	MZ357419	MZ357437	NA
*A.nigra*	CGMCC 3.16059	MZ354143	MZ350137	MZ357405	MZ357424	NA
*A.nigra*	CGMCC 3.16060	MZ354144	MZ350138	MZ357406	MZ357425	NA
*A.oblongispora*	CGMCC 3.16061	MZ354145	MZ350139	NA	MZ357426	NA
*A.ovalispora*	CGMCC 3.16019	NR_176748	MW264131	NA	NA	NA
*A.panacisoli*	SYPF 7183*	MF522181	MF522180	MF624251	NA	MF522179
*A.pararepens*	XY00631	OL620085	ON123774	NA	NA	NA
*A.pararepens*	XY00615	OL620086	NA	NA	NA	NA
*A.pararepens*	XY05899	OL620087	NA	NA	NA	NA
*A.pararepens*	CCF 6352	MT193669	MT192308	NA	NA	NA
*A.pernambucoensis*	URM < BRA > 7219	MN635568	MN635569	NA	NA	NA
*A.pseudocylindrospora*	EML-FSDY6-2	KU923817	KU923814	NA	KU923815	KU923819
*A.psychrophilia*	FSU4745	AY944874	EU736306	EU736252	AY944762	EU736279
*A.purpurea*	CGMCC 3.16106	OL678135	NA	NA	NA	NA
***A.pyriformis***	CGMCC 3.28538*	**PQ610531**	**PQ605102**	**PQ613267**	**PQ613277**	**PQ605112**
***A.pyriformis***	**XG09540-14-5**	**PQ610532**	**PQ605103**	**PQ613268**	**PQ613278**	**PQ605113**
*A.radiata*	CGMCC 3.16257	ON074698	ON074684	NA	NA	NA
*A.radiata*	XY09330-1	ON074699	ON074685	NA	NA	NA
*A.repens*	CBS 115583*	NR_103624	NG_058551	NA	NA	NA
*A.saloaensis*	URM 8209*	MN953781	MN953783	NA	NA	NA
*A.sichuanensis*	CGMCC 3.16258*	NR_182589	ON074688	NA	NA	NA
*A.soli*	MFLU-20-0414*	MT396373	MT393988	NA	NA	MT394049
*A.spinosa*	FSU551	AY944887	EU736307	EU736253	EU736227	EU736280
*A.stercoraria*	EML-DG8-1*	KU168828	KT921998	KT922002	KT922000	NG_065640
*A.sympodialis*	CGMCC 3.16063*	MZ354147	MZ350141	NA	NA	NA
*A.sympodialis*	CGMCC 3.16064	MZ354148	MZ350142	MZ357408	NA	NA
***A.tardiva***	CGMCC 3.28537*	**PQ610529**	**PQ605100**	**PQ613265**	**PQ613275**	**PQ605110**
***A.tardiva***	**XG08757-6**	**PQ610530**	**PQ605101**	**PQ613266**	**PQ613276**	**PQ605111**
*A.terrestris*	FMR 14989*	LT795003	LT795005	NA	NA	NA
***A.tibetensis***	CGMCC 3.28534*	**PQ610535**	**PQ605106**	**PQ613271**	**PQ613281**	**PQ605116**
***A.tibetensis***	**XG00415-3**	**PQ610536**	**PQ605107**	**PQ613272**	**PQ613282**	**PQ605117**
*A.turgida*	CGMCC 3.16032*	NR_189830	NG_241931	MZ357415	MZ357434	NA
*A.varians*	CGMCC 3.16065*	MZ354149	MZ350143	MZ357409	MZ357428	NA
*A.virescens*	CGMCC 3.16066*	MZ354150	MZ350144	MZ357410	MZ357429	NA
*A.virescens*	CGMCC 3.16067	MZ354151	MZ350145	MZ357411	MZ357430	NA
*A.xinjiangensis*	CGMCC 3.16107*	OL678136	NA	NA	NA	NA
*A.yunnanensis*	XY09528	ON074701	ON074686	NA	NA	NA
*A.yunnanensis*	CGMCC 3.16259*	NR_182591	NG_149054	NA	NA	NA
*A.zonata*	CGMCC 3.16033*	NR_189831	MW671548	MZ357416	MZ357435	NA
*A.zygospora*	RSPG 214	KC478527	NA	NA	NA	NA
*A.zygospora*	ANG28	DQ914420	NA	NA	NA	NA
*A.zygospora*	MFLUCC 23-0016*	OR104965	OR104992	NA	NA	NA
*Cunninghamellablakesleeana*	CBS 782.68	JN205869	MH870950	NA	NA	NA
*C.blakesleeana*	CBS 133.27*	JN205865.1	MH866397.1	KJ156479.1	NA	NA
*C.elegans*	CBS 167.53	MH857146	HM849700	NA	NA	NA
*C.elegans*	CBS 160.28*	AF254928.1	NA	KJ156470.1	NA	NA

Notes: The newly discovered species identified in the present study are in bold. Ex-type strains are marked with a star marker “*”. NA stands for “not available”.

*Absidia* has important physiological functions, which are manifested in many aspects, such as ecology, industry, medicine, and so on. Ecologically, it helps in the decomposition of organic matter, which is essential for nutrient cycling. Industrially, it is used for the biotransformation of various natural products. However, it also has a downside: some species of the genus *Absidia* that can grow at 37 °C are opportunistic pathogens that cause diseases in humans and animals (Zhao et al. 2022b; Tao et al. 2024). Therefore, there is still great research value in the physiological function of the genus *Absidia*. As of 11 November 2024, the Global Biodiversity Information Facility (GBIF) (https://www.gbif.org/, accessed 11 November 2024) contains 8,496 globally reported georeferenced records of the genus *Absidia* species. The genus is most widely distributed in Europe and least in Antarctica (Ellis and Hesseltine 1965; Tran et al. 2019; Liu et al. 2021). In this study, new species of *Absidia* were found in regions with different climates in Yunnan, Hainan, and Tibet in China, which further revealed the species diversity of *Absidia* in different regions.

## Supplementary Material

XML Treatment for Absidia collariata

XML Treatment for Absidia hainanensis

XML Treatment for Absidia pyriformis

XML Treatment for Absidia tardiva

XML Treatment for Absidia tibetensis

## ﻿Additional information

### Author ORCIDs

Xin-Yu Ji https://orcid.org/0009-0000-4121-9103

Zi-Ying Ding https://orcid.org/0009-0003-1618-5740

Yong Nie https://orcid.org/0000-0001-8964-1661

Heng Zhao https://orcid.org/0000-0003-2938-5613

Shi Wang https://orcid.org/0000-0002-7376-7638

Bo Huang https://orcid.org/0000-0001-6032-7396

Xiao-Yong Liu https://orcid.org/0000-0002-8808-010X

### Data availability

The sequences of this study have been submitted to the NCBI database (https://www.ncbi.nlm.nih.gov/, accessed November 15, 2024) with accession numbers shown in Table 2.
